# Hemodynamic response to high-volume hemofiltration in pediatric septic shock

**DOI:** 10.1016/j.jointm.2026.04.006

**Published:** 2026-06-06

**Authors:** Vladimir L. Cousin, Laura Ameloot, Luc Morin, Ramy Charbel, Philippe Durand, Laurent Chevret, Jordi Miatello, Pierre Tissières

**Affiliations:** 1 IHU-PROMETHEUS Comprehensive Sepsis Center, Pediatric Intensive Care, Neonatal Medicine and Pediatric Emergency Department, AP-HP Paris Saclay University, Bicêtre Hospital, Le Kremlin-Bicêtre, France; 2 Institute of Integrative Biology of the Cell, CNRS, CEA, Paris Saclay University, Gif-sur-Yvette, France; 3 Intensive Care, Mayotte Hospital, Mamoudzou, Mayotte, France; 4 Pediatric Department, Centre Hospitalier Yves le Foll, Saint Brieuc, France

**Keywords:** Pediatric, Refractory shock, Septic shock, High-volume hemofiltration, Vasoactive-inotropic score, Extracorporeal blood purification techniques

## Abstract

**Background:**

High-volume hemofiltration (HVHF) has recently been recommended as an adjunctive therapy in pediatric septic shock; however, limited data on its therapeutic impact and the absence of defined response criteria remain concerns for its utilization. We aimed to report the hemodynamic response, patient characteristics, and their association with outcomes in children with refractory septic shock responding to HVHF.

**Methods:**

We conducted a single-center cohort retrospective, descriptive, observational study of patients with septic shock receiving HVHF therapy. The progression of the vasoactive-inotropic score (VIS) from the start to 24 h of HVHF therapy was evaluated. The optimal timing and reduction of VIS were identified as predictors of survival, informing the most appropriate criteria for HVHF therapy responsiveness. Finally, the characteristics of patients who responded to HVHF were analyzed.

**Results:**

A total of 64 patients were included, with a median age of 26 months (interquartile range [IQR]: 7.5–82.0), all of whom were mechanically ventilated. The median VIS at inclusion was 411.2 (IQR: 168–770), and the median computed Septic Shock Score was 20.1 (IQR: 3.1–21.3). The median time from admission to HVHF initiation was 24 h (IQR: 12–48) and an ultrafiltrate rate of 80 mL/(kg·h) (IQR: 53–100). Overall, the survival rate was 27/64 (42.2%). The best response criterion was a >50% reduction in VIS after 12 h of HVHF therapy, with a survival rate of 65.2% (15/23) in responders *vs*. 32.4% (11/34) in nonresponders (*P*=0.010). Factors associated with response to HVHF included weight (odds ratio [OR]=1.04; 95% confidence interval [CI]: 1.00 to 1.08, *P*=0.040), age (OR=1.01; 95% CI: 1.00 to 1.02; *P*=0.280), and serum creatinine (OR=1.01; 95% CI: 1.00 to 1.02; *P*=0.046).

**Conclusions:**

Our study indicated that a reduction of over 50% in the VIS after 12 h of HVHF therapy is the most reliable predictor of survival in children with refractory septic shock. These hypothesis-generating criteria may assist in early assessment of HVHF response.

## Graphical abstract

**Figure:**
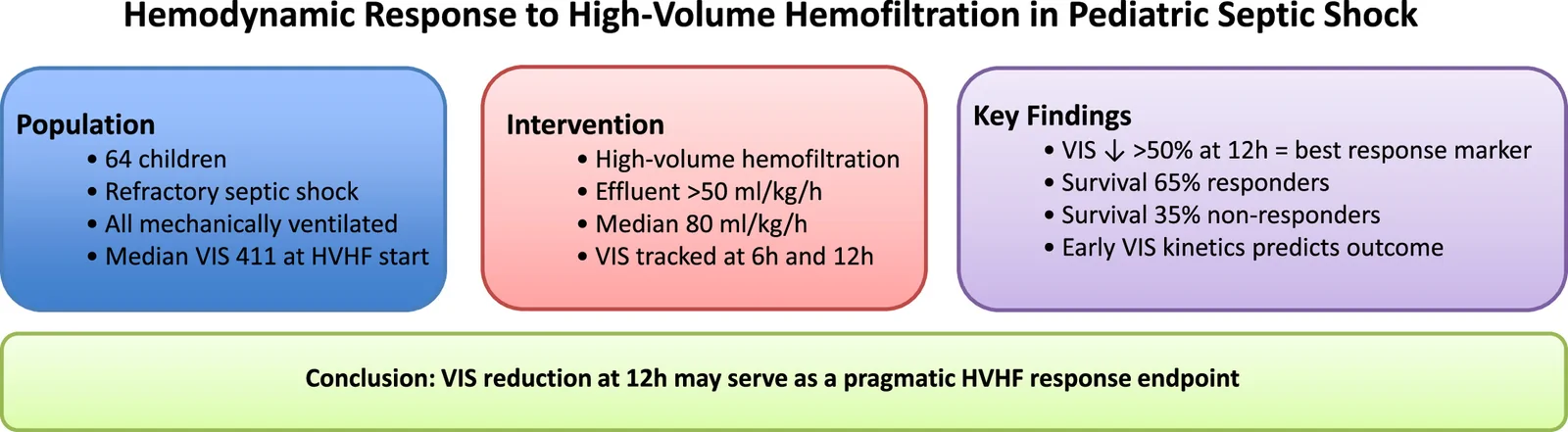

## Introduction

Sepsis remains a leading cause of pediatric morbidity and mortality worldwide.^[^^1^^]^ It is characterized by a dysregulated immune response, with imbalanced cytokine levels and vasoplegic circulating molecules, ultimately leading to multiple organ failure. Recently, extracorporeal blood purification techniques (EBPTs) have been suggested as adjunct therapies in addition to standard organ support and are aimed at removing circulating compounds from plasma and tissues. Nearly 75% of intensivists report using at least one adjunctive EBPT in their clinical routine.^[^^2^^]^ Several blood purification techniques exist, ranging from high-volume hemofiltration (HVHF) to adsorption methods.^[^[2], [3], [4], [5]^]^ Technologies used in blood purification therapies include high-plasma-volume treatment, high-cut-off membranes, and membranes with specific adsorption capacities.^[^^6^^]^ HVHF operates on the principle that high convection effluent flux enhances the removal of inflammatory cytokines and cellular by-products. Studies examining the use of HVHF have shown promising yet mixed results in both adults and children. In adults, several studies have emphasized the lack of clear benefits of HVHF in septic shock; however, a recent meta-analysis suggests a reduction in mortality.^[^[7], [8], [9]^]^ In children, a recent meta-analysis by Bhatt et al.^[^^10^^]^ indicates that HVHF might influence mortality, supporting longstanding clinical experiences of HVHF in various pediatric conditions such as macrophage activation syndrome, acute liver failure, and septic shock.^[^[11], [12], [13], [14]^]^ These studies underpin the recent recommendations by the pediatric Surviving Sepsis Campaign guideline for the use of HVHF in pediatric septic shock.^[^^15^^]^

The therapeutic response to HVHF has not yet been precisely measured and is not yet used in clinical practice, as most studies focus on patients’ survival. Pickkers et al.^[^^16^^]^ proposed that an early decrease in vasopressor support within 6 or 12 h of starting therapy could serve as a surrogate marker for a successful response to EBPTs. This brief delay in assessing responsiveness to EBPTs highlights the likely connection between therapy and hemodynamic effects. Recently, Medica et al.^[^^17^^]^ confirmed that the HVHF effect on cytokine clearance occurs within the first 12 h of therapy. Although hemodynamic stabilization, as demonstrated by the vasopressor sparing effect, could be considered a surrogate for septic shock treatment effectiveness,^[^^18^^]^ the absence of established criteria to evaluate the outcome of patients on HVHF leads us to investigate if the vasoactive-inotropic score (VIS) progression after initiating HVHF may serve as bystander of patient outcome in pediatric septic shock on HVHF.

## Methods

### Study design and patients

We conducted a retrospective observational study in the Pediatric Intensive Care Unit (PICU) at Bicêtre Hospital (AP-HP Paris-Saclay University, Le Kremlin-Bicêtre, France), over the period from 1 January 2003 to 31 December 2024 were identified through the hospital’s electronic database. In our unit, HVHF is used for septic shock, acute liver failure, acute kidney injury, and severe postoperative vasoplegic shock. Inclusion criteria comprised patients admitted to the unit with septic shock treated with HVHF. Exclusion criteria comprised patients who received HVHF for indications other than septic shock. The study (French study classification MR004) received approval from the French Society of Intensive Care Ethics Committee (CE SRLF 20-46). The requirement for written informed consent was waived.

### Study endpoints and data collection

The primary endpoint was 28-day mortality. Secondary endpoints included the degree of reduction in vasopressor support, the time delay to achieving that reduction, and the rate of positive response to HVHF. The study also aimed to describe patient characteristics associated with a positive response to HVHF and to assess the predictive value of that response for patient outcomes. We analyzed the hemodynamic response by examining the reduction of VIS at 6 and 12 h after starting HVHF, following the proposition of Pickkers et al.^[^^16^^,^^19^^]^

VIS=100 × (epinephrine + norepinephrine in µg/(kg·min)) + (dobutamine + dopamine in µg/(kg·min)) + 10,000 × (vasopressin in U/(kg·min)) + 10 × (milrinone in µg/(kg·min)).

We collected standard patient demographics, the primary indication for HVHF initiation, and laboratory test results at the start of therapy. Septic shock severity was reported using the Septic Shock Score (SSS), with both clinical SSS and bedside scores being calculated retrospectively.^[^^20^^]^

### High-volume hemofiltration procedure

In each case, the treatment was initiated and monitored by the attending physician responsible for the patient. Initiation of HVHF is the standard care in our unit for cases of refractory septic shock. We inserted a hemofiltration catheter (dual coaxial lumen catheter 6–12 Fr) percutaneously under ultrasound guidance using the Seldinger technique. Blood products, especially platelets, fibrinogen, or fresh frozen plasma, were administered if needed during catheter insertion to reduce the hemorrhagic risk. Two control units were used: Aquarius (Edwards Lifesciences, Guyancourt, France, 2003–2017) and Prismaflex (Baxter Acute Therapies, Meyzieu, France; 2017–2024), with appropriately selected membranes tailored to patient weight and the recommended surface area. Replacement solutions used were either Hemosol B0 or Phoxylium (both from Gambro Lundia AB, Lund, Sweden). All therapies were administered using hemodiafiltration modalities. Blood flow was adjusted to the maximum capacity of the catheter and the patient’s hemodynamic tolerance. A filtration fraction below 20% was maintained. The replacement solution was divided between the pre- and post-filter in ratios of 1:3 and 2:3, respectively. Extracorporeal circuits were primed with normal saline and unfractionated heparin. Continuous anticoagulation with unfractionated heparin during HVHF therapy was personalized based on each patient’s hemorrhagic risk, particularly in cases of coagulopathy. The duration of therapy was decided at the discretion of the attending physician.

### Statistical analysis

The demographic and clinical characteristics of patients were described using medians and interquartile ranges (IQRs) for continuous variables and proportions (%) for categorical variables. VIS variation from baseline was calculated as ([VIS at 6 or 12 h]/[VIS at initiation of HVHF]) ×100. To identify the optimal hemodynamic response criteria for 28-day mortality, the Youden index was calculated and presented as a heat map, with higher values indicating the optimal VIS ratio. Based on these criteria, comparisons between responders and non-responders were performed using the Mann–Whitney test for continuous variables and Fisher’s exact test for categorical variables. Comparison between VIS and lactate at different time points was performed using the Wilcoxon paired test. To evaluate predictors of outcome, logistic regression was used to estimate odds ratios (ORs) and 95% confidence intervals (CIs). We limited the use of multivariate analysis to the main outcomes due to the relatively small number of patients in our study, which limited its applicability. Statistical analyses were conducted using Stata software v18 (StataCorp, College Station, TX, USA) and GraphPad Prism software (GraphPad Version 9.0, GraphPad Software, San Diego, CA, USA).

## Results

During the study period, 223 patients received HVHF treatment; after excluding those treated for indications other than septic shock – including acute liver failure (*n*=88), acute kidney injury (*n*=23), non-septic vasoplegia (e.g., post-liver transplant ischemia-reperfusion syndrome or rhabdomyolysis; *n*=20), and cases with missing outcome data (*n*=27) – a total of 64 patients were included in the final population. Patients’ demographic characteristics are summarized in Table 1. At inclusion, all patients were in shock, intubated and mechanically ventilated, treated with vasopressors with a median VIS of 411.2 (IQR: 169–646), and 62.5% (40/64) received inotropic support for cardiac failure. Median arterial blood lactate was 5.1mmol/L (IQR: 2.8–8.8). The median bedside SSS was 3 (IQR: 1–4), and the median computed SSS was 20.5 (IQR: 3.1–21.3). Sixty-two patients were treated by norepinephrine (96.9%), 18 patients were on epinephrine, 8 patients on vasopressin, and 4 patients on milrinone or 19 patients on dobutamine, a total of 35 patients having a cardiac dysfunction (Table 1). Accordingly, 47 patients were in refractory shock according to the European Society of Paediatric and Neonatal Intensive Care definition with computed SSS ≥3.5.^[^^20^^]^ Gram-negative bacteria were the most common pathogens identified (31/64, 48.4%), followed by Gram-positive bacteria (10/64, 15.6%), and viruses or parasites (11/64, 17.2%). A substantial proportion of patients had underlying liver disease (26/64, 40.6%), including 11 who had undergone liver transplantation. The time from admission to HVHF initiation was 24 h (IQR: 12–48), and all patients had an ultrafiltration flow rate exceeding 50 mL/(kg·h), with a median of 80 mL/(kg·h) (IQR 53–100). Overall, the survival rate was 42.2%. The kinetics of VIS and lactate during HVHF are shown in Figure 1, with a significant decrease in both VIS and lactate in survivors, as early as after 6 h of treatment, suggesting that early response is clinically relevant.

**Table 1:** Patients’ characteristics at HVHF initiation (*n*=64).

Characteristics	Data
Age (months)	26.0 (7.5–82.0)
Weight (kg)	13.0 (8.0–25.5)
PELOD score	21 (11–23)
PRISM Ⅱ score	16 (10–20)
Sex (male)	37 (57.8)
Liver disease	26 (40.6)
Post liver transplantation	11 (17.2)
Computed SSS	20.1 (3.1–21.3)
Bedside SSS	3.0 (1.0–4.0)
Refractory septic shock (clinical SSS≥3.5)	47 (73.4)
Cardiac dysfunction	35 (54.7)
Total leucocytes (×10^9^/L)	11.49 (5.09–23.53)
Serum creatinine (µmol/L)	90 (56–139)
Serum total bilirubin (mmol/L)	32 (7–116)
Hydrocortisone	60 (93.8)
Norepinephrine tartrate	62 (96.8)
Epinephrine	18 (28.1)
Vasopressin	8 (12.5)
Milrinone	4 (6.3)
Dobutamine	8 (12.5)
Survival rate	27 (42.2)

Data are expressed as median (interquartile range) or *n* (%).

HVHF, High volume hemofiltration; PELOD, Pediatric logistic organ dysfunction; PRISM, Pediatric risk of mortality; SSS, Septic shock score.

**Figure 1:**
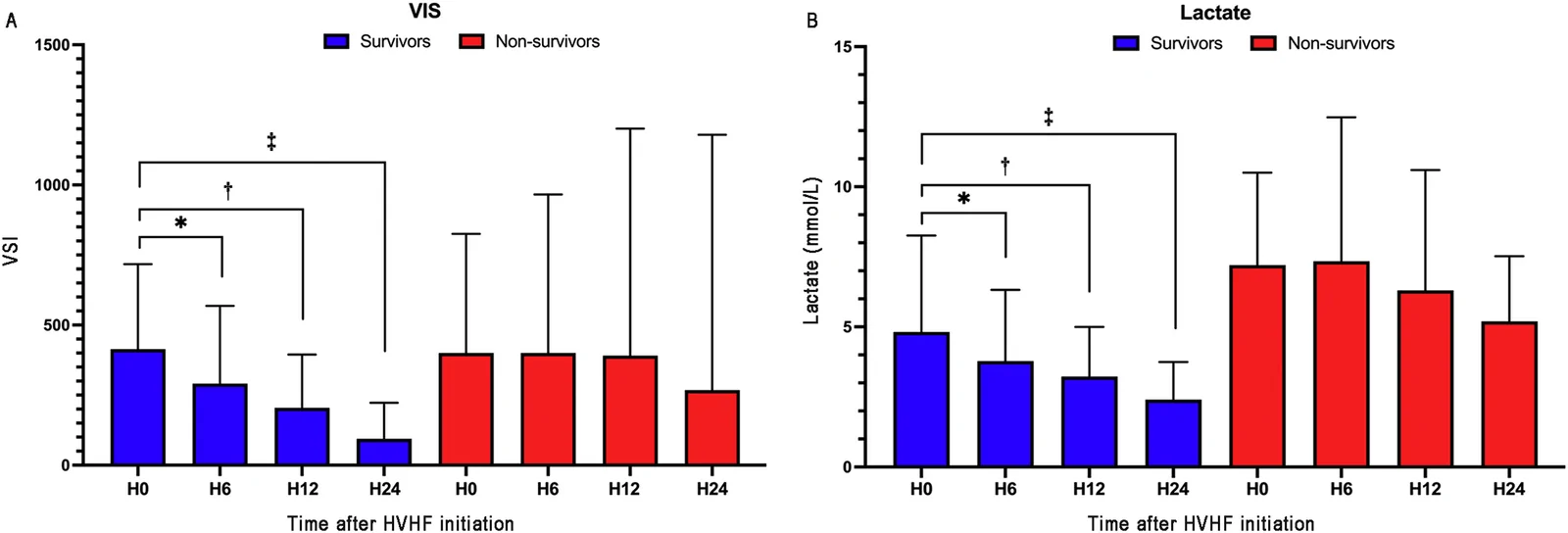
Kinetics of VIS and arterial blood lactate to HVHF. Kinetics of VIS (A) and lactate (B), according to the survival status. The *x*-axis stands for the time after HVHF initiation and the *y*-axis for VIS (A) or lactate (mmol/L) (B). Evolution of VIS showed significant reduction between H0 and H6 (*P=0.020), H0 and H12 (^†^*P*=0.010), H0 and H24 (^‡^*P* <0.001). Lactate evolution showed a significant reduction between H0 and H6 (*P=0.030), H0 and H12 (^†^*P*=0.010), H0 and H24 (^‡^*P*=0.001). H, Hour from HVHF start; HVHF, High-volume hemofiltration; VIS, Vasoactive-inotropic score.

To evaluate the hemodynamic response to HVHF, we monitored serial changes in the VIS. The median VIS was 411.2 (IQR: 168.0–770.0) at initiation of HVHF, 358.4 (IQR: 102.0–769.0) at 6 h, 263.8 (IQR: 90.0–700.0) at 12 h, and 132.5 (IQR: 59.2–347.5) at 24 h after therapy initiation. A 50% decrease in VIS at 12 h after HVHF initiation was the strongest independent predictor of survival, as illustrated in Figure 2. A decrease of 50% in VIS at 12 h yielded an area under the receiver operating curve of 0.64 (95% CI: 0.52 to 0.77) (Youden Index 0.28), being the most reliable early indicator for identifying a hemodynamic response to HFHV in septic shock. Based on the hemodynamic response criteria for HVHF, patients were categorized as responders or non-responders. Their characteristics are summarized in Table 2 and Supplementary Table S1. Notably, although responders exhibited more severely disease, as indicated by a higher prevalence of myocardial dysfunction (41.2% *vs*. 73.9%, *P*=0.030), they demonstrated a higher survival rate (15/23, 65.2%) compared with non-responders (11/34, 32.4%; *P*=0.010). Being a responder showed an OR of 3.90 (95% CI: 1.28 to 12.00, *P*=0.010) for survival (Table 3). Multivariate model for survival prediction, incorporating age, serum creatinine, presence of liver disease, and response status showed a persistent significant protective impact of being a responder (OR=5.80, 95% CI: 1.10 to 30.90, *P*=0.038).

**Figure 2:**
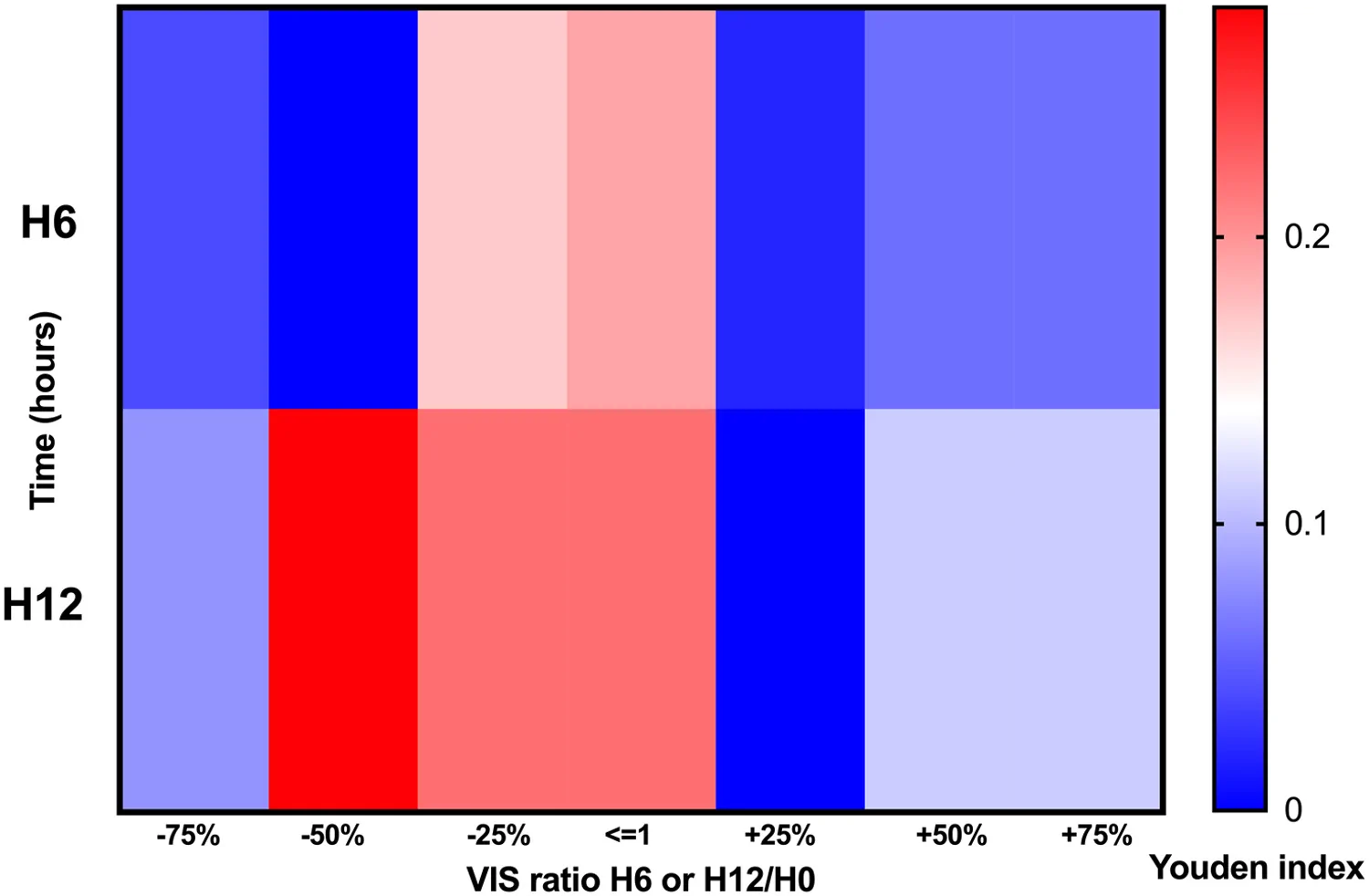
Hemodynamic response criteria to HVHF. Heatmap representing the calculated Youden index based on the receiver operating characteristic curve established with variable VIS at 6 and 12 h after HVHF treatment, predicting 28-day survival. Best predictive accuracy (red) compared to worst accuracy (dark blue). H: Hour from HVHF start; HVHF, High-volume hemofiltration; VIS, Vasoactive-inotropic score.

**Table 2:** Patients’ characteristics according to hemodynamic responsiveness to HVHF therapy.*^,^†

Characteristics	Non-responder (*n*=34)	Responder (*n*=23)	*P* value
Age (months)	14 (7–44)	46 (12–130)	0.030
Weight (kg)	10.8 (7.0–15.0)	18.0 (10.0–33.8)	0.006
Computed SSS‡	4.9 (3.0–20.9)	20.6 (4.1–21.2)	0.140
Bedside SSS‡	2 (1–4)	4 (1–4)	0.190
Refractory septic shock (cSSS ≥ 3.5)	23 (67.6)	18 (78.3)	0.380
Cardiac dysfunction	14 (41.2)	17 (73.9)	0.030
Total leucocytes (×10^9^/L)‡	9.85 (4.31–24.60)	16.68 (10.78–23.75)	0.150
Serum creatinine (µmol/L)‡	67.0 (53.0–105.0)	125.0 (85.5–191.5)	0.004
Total serum bilirubin (mmol/L)‡	53.5 (16.0–308.5)	8.5 (6.0–74.5)	0.040
Arterial blood lactate (mmol/L)‡	4.6 (3.0–7.7)	4.5 (2.5–8.1)	0.580
VIS‡	400.0 (168.0–750.0)	411.2 (160.0–550.0)	0.650
Norepinephrine tartrate dose (µg/(kg·min))‡	3.69 (1.66–7.57)	4.00 (0.74–5.50)	0.440
Ultrafiltration rate (mL/(kg·h))	81.5 (64.0–94.0)	62.0 (50.0–100.0)	0.380
Associated liver disease	16 (47.1)	6 (26.1)	0.160
Survival	11 (32.4)	15 (65.2)	0.010

Data are expressed as median and interquartile range (IQR) or *n* (%).

HVHF, High volume hemofiltration; SSS, Septic Shock Score; VIS, Vasoactive-inotropic score.

⁎ Responsiveness is defined as a decrease in VIS by >50% by 12 h after HVHF initiation.

† Seven patients were excluded from the analysis due to early death to mitigate immortal time bias.

‡ At HVHF initiation.

**Table 3:** Logistic regression of patients’ characteristics according to survival status and incorporating the hemodynamic responsiveness to HVHF therapy.*

Characteristics	Non-survivor (*n*=31)	Survivor (*n*=26)	OR (95% CI)	*P* value
Age (months)	13 (7–32)	52 (9–130)	1.01 (1.00–1.02)	0.028
Weight (kg)	10.0 (7.6–16.0)	18.0 (10.0–30.0)	1.04 (1.00–1.08)	0.040
Computed SSS†	6.6 (3.2–21.1)	20.5 (2.8–21.0)	1.03 (0.98–1.09)	0.200
Bedside SSS†	2.0 (1.0–4.0)	3.5 (1.0–4.0)	1.20 (0.87–1.80)	0.220
Refractory septic shock (cSSS ≥ 3.5)†	23 (74.2)	18 (69.2)	0.78 (0.24–2.50)	0.680
Cardiac dysfunction†	13 (41.9)	18 (69.2)	3.10 (1.04–9.30)	0.040
Total leucocytes (10^9^/L)†	11.8 (4.8–24.6)	13.2 (6.5–23.7)	1.01 (0.98–1.04)	0.490
Serum creatinine (µmol/L)†	84 (66–105)	128.5 (52–206.5)	1.01 (1.0–1.02)	0.046
Total serum bilirubin (mmol/L)†	49 (12–272)	9 (6–62)	0.99 (0.99–1.00)	0.240
Arterial blood lactate (mmol/L)†	6 (3–8.9)	4.1 (2.5–5.8)	0.89 (0.77–1.03)	0.140
VIS†	483.8 (80–770)	357.9 (194.4–550)	0.99 (0.99–1.00)	0.980
Norepinephrine tartrate dose (µg/(kg·min))†	4.8 (0.74–8.8)	3.5 (1.7–5.5)	0.97 (0.9–1.05)	0.540
Ultrafiltration rate (mL/(kg·h))	76 (52–90)	90 (53–107)	1.01 (0.99–1.03)	0.190
Associated liver disease	17 (54.8)	5 (19.2)	0.19 (0.06–0.65)	0.008
Responder status‡	8 (25.8)	15 (57.7)	3.90 (1.28–12.00)	0.017

Data are expressed as median (interquartile range) or *n* (%).

CI, Confidence interval; HVHF, High volume hemofiltration; OR, Odds ratio; SSS, Septic Shock Score; VIS, Vasoactive-inotropic score.

⁎ Seven patients were excluded from the analysis due to early death to mitigate immortal time bias.

† At HVHF initiation.

‡ Responsiveness is defined as a decrease in VIS by >50% by 12 h after HVHF initiation.

## Discussion

We showed that a decline of >50% in the VIS, 12 h after HVHF initiation, may serve as a surrogate marker of the patient outcome in pediatric septic shock. This cut-off may therefore serve as a favorable hemodynamic response criterion to HVHF therapy in pediatric septic shock.

Despite a consistent decline in infection-related mortality in high-income countries, mortality from septic shock remains high worldwide, and variability in case mix is known to significantly impact outcomes.^[^^1^^]^ The most vulnerable patients with septic shock, exhibiting severe metabolic and circulatory failure, tend to have the poorest outcomes even with organ support and therapeutic escalation. A multicenter cohort from high-income countries comprising 534 children with septic shock reported a mortality rate of 14% and identified two severity groups: one with a 2.2% mortality rate and another, described as refractory septic shock, with a mortality of 60.3%, despite the use of organ support therapies (e.g., mechanical ventilation, continuous renal replacement therapy, extracorporeal membrane oxygenation).^[^^20^^]^ The refractory SSS was developed and validated based on three factors: serum lactate level, presence of myocardial dysfunction, and the level of vasoactive-inotropic support. This score was further validated in a middle-income country cohort, demonstrating similar accuracy (area under the ROC curve 0.91–0.95), where refractory septic shock was associated with a mortality rate of 85.7%.^[^^21^^]^ The benefits of adjunctive therapies are inherently linked to disease severity at the time of intervention and should be carefully assessed.^[^^22^^]^ The current use of HVHF, as an adjunct therapy, is reported to be utilized in the most severe patients with an associated mortality rate of 28–34%.^[^[23], [24], [25], [26]^]^ In our cohort, most patients had refractory septic shock and a mortality rate of 42%.

In addition to supporting renal function and homeostasis, the rationale for using HVHF during septic shock depends on its ability to remove cytokines and circulating vasoactive substances through increased convective capacities.^[^^4^^,^^27^^]^ Whereas the impact of HVHF on cytokine clearance in adult patients remains controversial, studies in children have more consistently reported a reduction in serum cytokine levels.^[^^25^^,^^26^^]^ Efficacy of convection depends on the extent of treated volume, with ultrafiltrate flow higher than 50 mL/(kg·h) defining HVHF.^[^[28], [29], [30]^]^ Three studies reporting HVHF treatment in pediatric septic shock described similar ultrafiltrate flow ranging between 50 and 60 mL/(kg·h).^[^[23], [24], [25]^]^ Accordingly, we reported the use of effluent flow exceeding 50 mL/(kg·h).

Despite initially promising results, the impact of HVHF on outcomes remains controversial.^[^^31^^]^ In adults with septic shock, the multicenter controlled IVOIRE study failed to confirm a beneficial effect on mortality.^[^^9^^,^^32^^]^ However, the most recent meta-analysis by Snow et al.^[^^8^^]^ found a mortality benefit of HVHF in septic shock, although additional data are needed, as shown by trial sequential analysis. In pediatric studies on HVHF, a recent meta-analysis pooling available studies found that, with three randomized controlled trails, HVHF seemed to have a favorable effect on patient mortality.^[^^10^^]^ A detailed analysis of existing experience indicates that the positive impact of HVHF may be related to different patient phenotypes.^[^^29^^,^^33^^,^^34^^]^ Importantly, all those studies compared HVHF to standard continuous renal replacement therapy flux, usually prescribed at 30 mL/(kg·h). Considering these cumulative experiences with HVHF in septic shock, the latest French guidelines on renal replacement therapy recommend the use of HVHF, as do the recent 2026 Surviving Sepsis Campaign pediatric guidelines, which advocate using HVHF over standard volume, despite a low certainty of evidence.^[^^15^^]^

Response criteria for HVHF remain poorly defined, despite their crucial clinical significance for patient selection and the choice of endpoints in future controlled trials. Using outcomes other than patients’ survival may provide more granularity regarding therapy effectiveness.^[^^35^^]^ Morin et al.^[^^13^^]^ reported that rapid improvement of vasopressor support can be observed with EBPT in children. Similarly, in adults, Boussekey et al.^[^^29^^]^ suggest that adding HVHF induces a swift and early reduction of vasopressive support, especially in patient subsets identified as responders. In our study, weaning from vasoactive-inotropic support was significantly linked to 28-day survival, supporting the idea that a better VIS response in the initial hours of therapy may serve as a response criterion for HVHF. Our findings identify a 50% decrease in VIS within 12 h of initiation may be a surrogate for patients’ outcome while on HVHF, confirming the suggestion by experts’ recommendation on EBPT.^[^^3^^,^^16^^]^

Our study is a retrospective, single-center investigation with all its inherent limitations. Moreover, the study period is prolonged, with inherent changes in practices regarding the selection of aminergic drugs, the expanded use of ultrasound, and changes in HVHF machines, with newer machines being more reliable and easier to use. We used a pragmatic approach, supported by extensive literature, to identify the most accurate hemodynamic response criteria for HVHF in children with septic shock. While these criteria require further validation, they can already serve as surrogate endpoints for assessing patient outcomes while on HVHF. Improved identification of patient phenotypes remains essential, as hemodynamic improvement may reflect the impact of a complex bundle of care for septic shock that incorporates HVHF, rather than solely the use of HVHF. Our data do not provide sufficient patient characteristics to identify responders at the outset and may necessitate advanced phenotyping that integrates detailed clinical, transcriptomic, and proteo-lipidomic signatures. Moreover, our data were not designed to provide a mechanistic explanation of the impact of HVHF.

To the best of our knowledge, this is the first study attempting to establish a clinical response criterion for HVHF, a crucial step in determining pragmatic outcomes for future research on HVHF and EBPT in pediatric septic shock.

## Conclusions

This study demonstrates that, among children receiving HVHF for refractory septic shock, a >50% reduction in the VIS at 12 h after HVHF initiation is significantly associated with improved survival. These parameters may serve as an early prognostic marker, giving substantial material for future validating controlled study.

## CRediT authorship contribution statement

**Vladimir L. Cousin:** Writing – review & editing, Writing – original draft, Visualization, Validation, Methodology, Formal analysis, Data curation, Conceptualization. **Laura Ameloot:** Writing – review & editing, Formal analysis, Data curation. **Luc Morin:** Writing – review & editing, Visualization, Validation, Formal analysis. **Ramy Charbel:** Writing – original draft, Validation, Formal analysis. **Philippe Durand:** Writing – review & editing, Visualization, Conceptualization. **Laurent Chevret:** Writing – review & editing, Validation, Data curation, Conceptualization. **Jordi Miatello:** Writing – review & editing, Validation. **Pierre Tissières:** Writing – review & editing, Writing – original draft, Visualization, Validation, Project administration, Methodology, Investigation, Formal analysis, Conceptualization.

## Appendix Supplementary materials

Image, application 1
