# Supplementary material for: Hemodynamic response to high-volume hemofiltration in pediatric septic shock
Source: J Intensive Med. 2026 Jun 6;6(5):517–23. doi: 10.1016/j.jointm.2026.04.006 (PMC13728384; doi:10.1016/j.jointm.2026.04.006)
Supplement: Supplementary file 1 [file mmc1.docx]

**Supplementary File**

**Supplementary Table S1. Predictive factors for haemodynamic response to HVHF therapy ***

|  | **OR (95% CI)** | ***p value*** |
| --- | --- | --- |
| **Sensitivity analysis 1** |  |  |
| Age (months) | 1.02 (1.00-1.02) | *0.045* |
| Weight (Kg) | 1.04 (1.00-1.09) | *0.03* |
| Computed SSS ^#^ | 1.04 (0.99-1.1) | *0.109* |
| Bedside SSS ^#^ | 1.28 (0.89-1.86) | *0.178* |
| Total leucocytes ^#^ (G/L) | 1.01 (0.98-1.05) | *0.348* |
| Serum creatinin (μg/kg/min) ^#^ | 1.01 (1.00-1.02) | *0.018* |
| Total serum bilirubin (mmol/l) ^#^ | 0.99 (0.99-1.00) | *0.216* |
| Arterial blood lactate (mmol/L) ^#^ | 0.98 (0.87-1.1) | *0.784* |
| Vaso-inotropic score ^#^ | 0.99 (0.99-1.00) | *0.611* |
| Norepinephrine dose (μg/kg/min) ^#, ¶^ | 0.95 (0.87-1.04) | *0.346* |
| Ultrafiltration rate (ml/kg/h) | 0.98 (0.97-1.01) | *0.298* |
| Associated liver disease | 0.39 (0.12-1.25) | *0.115* |
| **Sensitivity analysis 2** |  |  |
| Weight >15 kg | 4.5 (1.5-13.9) | *0.009* |
| Age 24-120 months | 3.9 (1.1-13.8) | *0.03* |
| Age >121 months | 4.7 (1-21.6) | *0.04* |
| Age >25 months | 4.2 (1.3-13) | *0.01* |
| Serum creatinine >90 mmol/L | 4.1 (1.2-14.6) | *0.027* |

* Responsiveness is defined as a decrease in VIS by more than 50% by 12 hours after HVHF initiation, ^#^ at HVHF start, ¶ norepinephrine tartrate.

HVHF, high volume hemofiltration; OR, odd ratio; CI, confidence interval; SSS, septic shock score
